# Respectful focused antenatal care and associated factors among pregnant women who visit Shashemene town public hospitals, Oromia region, Ethiopia: a cross‐sectional study

**DOI:** 10.1186/s12905-021-01237-0

**Published:** 2021-03-04

**Authors:** Daniel Adane, Agegnehu Bante, Biresaw Wassihun

**Affiliations:** 1 Department of Midwifery, College of Medicine and Health Sciences, Wolkite University, Wolkite, Ethiopia; 2 Department of Midwifery, College of Medicine and Health Sciences, Arbaminch University, Arbaminch, Ethiopia; 3 Department of Nursing, College of Medicine and Health Sciences, Arbaminch University, Arbaminch, Ethiopia

**Keywords:** Focused antenatal care, Respectful care, Ethiopia

## Abstract

**Background:**

Focused antenatal care is the most significant and inclusive care given to pregnant women to promote and maintain the optimal health of the mother and the fetus. Providing respectful care during focused antenatal care is believed to be the most important cost-effective interventions to increase maternity service utilization. Therefore, this study was aimed to assess respectful focused antenatal care and associated factors among pregnant women who visit Shashemene town public hospitals, Oromia region, Ethiopia, 2019.

**Methods:**

Institution-based cross-sectional study was conducted from July-August, 2019. A total of 423 pregnant mothers were selected using a systematic sampling technique. Data were collected using structured and pre-tested interviewer-administered questionnaires. Data entry and analysis were made using Epi Info version™ 7 and Statistical Package for Social Science (SPSS) version 24.0 respectively. Both bivariate and multivariate logistic regression analyses were used to identify associated factors. Statistical significance was declared at a *p* value of < 0.05 with a 95% confidence level.

**Results:**

A total of 420 women have participated in the study, making a response rate of 99.5%. About 63% of participants received respectful care during focused antenatal care. Having no formal education [AOR = 8.3(95%CI 9.85–17.47)], low average monthly income [AOR = 3.16 (95%CI 1.52–6.57)], having unplanned pregnancy [AOR = 9.90 (95%CI 3.48–8.16)] and being multigravida [AOR = 8.82 (95%CI 2.90–6.80)] were significantly associated with respectful focused antenatal care.

**Conclusions:**

The study findings indicate that respondents’ respectful focused antenatal care is mainly affected by educational level, average family monthly income, having an unplanned pregnancy, and gravidity. Providing acceptable, quality, and honorable care for all women regardless of educational status, family income, and status of pregnancy is very crucial to entice more mothers to the health facility.

**Supplementary Information:**

The online version contains supplementary material available at 10.1186/s12905-021-01237-0.

## Background

Improving maternal health is one of the recommendations to achieve the global target of sustainable development goals (SDGs) for the reduction of maternal mortality to less than 70 out of 100,000 live births [1]. Worldwide maternal morbidity and mortality have still been continued to be the major public health problems [2]. Globally, approximately three million women died from pregnancy and childbirth-related complications. Of these, all most all (99%) maternal death occurs in Low-and middle-income countries and nearly two-thirds (66%) of maternal deaths are more prevalent in sub-Saharan African countries [3]. In Ethiopia, the maternal mortality ratio was 412/100,000 live births [2, 4]. According to the Ethiopian demographic health survey (EDHS) 2016, the utilization of antenatal care (ANC) and skilled delivery were found to be 62% and 26% respectively. One of the main reasons for the low utilization of maternity services was disrespect and abuse by caregivers [5].

Pregnancy is an important event from both social and medical points of view but its related complications were a major cause of maternal death [6]. Obstetric abuse during antenatal care is one of the contributing factors for low maternal health care service utilization [7]. Antenatal care (ANC) is care given to pregnant women to promote and maintain optimal health of the mother and fetus throughout the pregnancy, labor, and puerperium [8]. A lack of respectful care during ANC visit is one indicator of poor quality of care [9]. Focused antenatal care (FANC) stated that every pregnant woman is at risk, so providing FANC is a good opportunity to identify preexisting and newly occurring diseases [2].

Respectful maternity care (RMC) is crucial to the health, wellbeing, and survival of women and newborns and it is also very important to fascinate more mothers to the health facilities [7, 10]. Although FANC is effective and has been widely promoted, data on respectful maternity care during focused antenatal care in study setting is limited; therefore this study was aimed to assess the level of respectful focused antenatal care and associated factors among pregnant women who visit Shashemene town public hospitals, Oromia region, Ethiopia.

## Methods

### Study design, setting, and subjects

An institution-based cross-sectional study was conducted in Shashemene town public hospitals from July-August 2019. Shashemene is located in the central part of the Oromia region, 248 kilometers far from Addis Ababa (the capital city of Ethiopia). The town has a total population of 218,335 from this nearly half (48.9%) of them were females. It consists of peoples with different languages, more than 18 ethnic groups, and foreigners like the Ras Teferian community. All pregnant women who were found in Shashemene town were the source population whereas all pregnant women who were attending ANC service in each hospital during the study period were taken as the study population. The town has two hospitals that provide ANC services and both hospitals were included in this study. The study populations were proportionally allocated to each hospital based on the average number of clients who received antenatal care services (279 from Kuyera Hospital and 144 from Melka odda Hospital).

### Sample size determination and techniques

The minimum sample size was determined using a single population proportion formula and it was calculated using Epi Info™ version 7 based on the following assumptions. An estimated level of respectful and abuse-free maternity care during antenatal care 50% (P = 0.5), 95% confidence level, and a margin error to be 5% (d = 0.05) and 10% non-response rate. The final sample size was therefore calculated to be 423 pregnant mothers. A systematic random sampling technique was used to select sampled pregnant mothers that represent the entire hospital. The interval, K = 4, was calculated by dividing the total number of ANC follow up mothers based on those mothers who received antenatal care services at each health facility in the month proceeding the data collection period. Then the first client was selected by simple random sampling among the first four antenatal care followers in the sampling frame. The data collection was begun after informing the related local authorities about the objectives of the study. Data collectors were worked with doctors and midwives in the ANC outpatient wards every day until the desired sample sizes were achieved. Every 4th pregnant mothers who come to ANC outpatient departments (OPDs) for routine antenatal care follow-ups were selected in each hospital. After informing and obtaining informed written consent, the trained data collectors interviewed eligible and randomly selected mothers in each ANC ward.

### Data collection and measurements

A structured and pre-tested interviewer-administered questionnaire was prepared by reviewing relevant works of different literature. The tool was adopted and modified into the local context from previously published articles, and WHO guidelines. Four experienced BSc midwives and two supervisors were recruited and trained for data collection and supervision, respectively. The questions were classified into socio-demographic characteristics, obstetric history, and respectful related items. Data were collected through the face-to-face interview method. The supervisors and principal investigators supervised the process of data collection daily. Respondents who responded correctly (yes) at least ≥ 10 questions from 15 respectful items were considered as having respectful care, whereas those respondents who have reported at least two “No” questions from 15 respectful items were considered as having no respectful care. The values of respectful FANC were coded as “1 = Correct response (consistent with respectful components) and 0 = Incorrect response (inconsistent with respectful care components)”. Finally, a composite variable from these questions was generated to categorize pregnant women as having “respectful care/abusive care.

To ensure quality, the questionnaire was translated into the local language by experts. Finally, before data collection, it was re-translated back to English to verify consistency. Before starting the actual data collection, one day of extensive training was given for the data collectors and supervisors. A pre-test for appropriateness and feasibility of the tool was conducted and all necessary modifications and amendments were done accordingly. The tool was used with a reliability test or Cronbach’s alpha correlation coefficient of greater than or equal to 0.7 for inter-item consistency. The data collection team was communicated and discussed with principal investigators if they face any challenges during the data collection period daily. After data collection before analysis, all collected data were checked for completeness.

### Statistical analyses

Data were coded, cleaned, edited, and entered into Epi Info version™ 7 and exported to SPSS version 24.0 for statistical analysis. The presence of an association between explanatory and outcome variables was ascertained using binary logistic regression analysis. The goodness of fit was tested by the log-likelihood ratio (LR). To control all possible confounders all variables with P < 0.2 in the bivariate analysis were included in the final model of multivariable analysis. To see the correlation between independent variables Multi-collinearity test was carried out by using collinearity statistics. In a multivariable model adjusted odds ratio determined with a 95% confidence level was used to assess the strength of association. In this study *p* value < 0.05 was deemed to declare statistical significance. Then, the finding was presented by using simple frequencies, summary measures, tables, texts, and figures.

## Results

### Socio‐demographic characteristics of respondents

In this study, a total of 420 respondents have participated with a response rate of 99.5%. The mean age of study participants was 25.8 (SD ± 3.7 years). More than half, 224 (53.3%) of respondents were in the age group of 26–30 years. Two hundred thirty-three (55.5%) were orthodox Christian religious followers. One hundred ninety-four (46.2%) of the respondents had a formal education, and 56.0% of the respondents have been working as a housewife (Table 1).

**Table 1:** Socio-demographic characteristics of respondents at Shashemene town hospitals, 2019 (n = 420)

Variables	Category	Frequency	Percentage
Age	20–25	181	43.1
	26–30	224	53.3
	31–35	15	3.6
Marital status	Married	370	87.7
	Divorced	19	4.5
	Widowed	20	4.7
	Single	11	2.6
Religion	Orthodox	233	55.5
	Muslim	73	17.4
	Protestant	114	27.1
Ethnicity	Oromo	310	73.8%
	Amhara	90	21.4%
	Tigre	8	1.9%
	Wolyita and Sidama	12	2.9%
Occupation	Housewife	235	56.0
	Private employee	15	3.6
	Government employee	84	20.0
	Merchants	57	13.6
	Daily workers	29	6.9
Residence	Urban	227	54.0
	Rural	193	46.0
Educational status of mothers	No formal education	194	46.2
	Primary (1–8)	58	13.8
	Secondary (9–12)	42	10.0
	Collage and above	126	30.0
Husbands educational status	No formal education	99	23.6
	Primary and secondary	122	29.0
	Collage and above	199	47.4
Average family monthly income	< 3000 birr	237	56.4
	≥ 3000 birr	183	43.6
Family size	1–3	157	37.4
	>3	263	62.6

### Obstetric characteristics of respondents

Nearly half (49.8%) of respondents were Primiparous and the majority (96.7%) of the respondents had a history of antenatal care (ANC) follow-up. Four hundred six (96.7%) of respondents had supported/planned pregnancy. More than three-fifth (62.6%) of the respondents had a previous history of institutional delivery and 90.4% of respondents have planned to give birth in health facilities (Table 2).

**Table 2:** Obstetric characteristics of respondents at Shashemene town hospitals, 2019 (n = 420)

Variables	Category	Frequency	Percentage
Gravidity	Prima- gravid	114	27.1
	Multi gravid	306	71.9
Parity	Primiparous	209	49.8
	Multiparous	211	50.2
History of abortion	Yes	20	4.8
	No	400	95.2
History of stillbirth	Yes	14	3.3
	No	406	96.7
The previous history of antenatal care follow up	Yes	406	96.7
	No	14	3.3
Frequency of current antenatal care visit	1st	45	10.7
	2nd	200	47.6
	3rd	147	35.0
	4th	28	6.7
Pregnancy status	Planned	406	96.7
	Unplanned	14	3.3
History of health facility delivery	Yes	263	62.6
	No	157	37.4
Do you have a plan to give birth in a health facility’s	Yes	380	90.4
	No	40	9.6
If no why (n = 40)	Home is better	20	50.0
	Fear of health care providers	8	20.0
	Not satisfied with antenatal care	5	12.5
	No reason	7	17.5

### Prevalence of respectful focused antenatal care

**Fig. 1:**
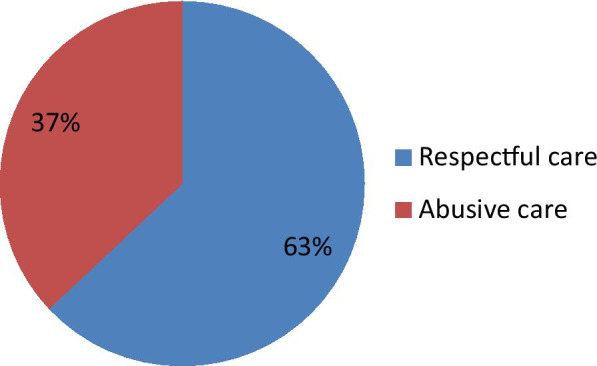
prevalence of respectful focused antenatal care at Shashemene town public hospitals, 2019 (n = 420)

Three hundred ninety-two (93.3%) of respondents reported that health care providers were explained procedures by giving greetings before the examination. Nearly half (46.7%) of respondents experienced obstetric violence/abuse during focused antenatal care counseling on birth preparedness and complication readiness plan and 139 (33.1%) of respondents reported that their waiting time for examination was not fair (Table 3). About 37% of respondents reported having experienced at least one form of abuse during focused antenatal care (Fig. 1).

**Table 3:** Prevalence of respectful focused antenatal care at Shashemene town hospitals, 2019 (n = 420)

Respectful/client-centered items during the focused antenatal care visit	Yes	No
Did the health care provider respect your culture and religion during the general examination	392 (93.3)	28 (6.7)
Did the health care provider explain procedures by giving a greeting before the examination	392 (93.3)	28 (6.7)
Did the health care provider treated you in a friendly manner	378 (90)	42 (10)
Did the Health care provider showed his/her concern and empathy	392 (93.3)	28 (6.7)
Did the Health care provider explained types of laboratory investigation in a satisfactory way	378 (90)	42 (10)
Did health care provider caring for you with a kind approach by calling your name	406 (96.7)	14 (3.3)
Did the health care provider responded to your needs whether or not you asked during counseling on birth preparedness and complication readiness	224 (53.3)	196 (46.7)
Did the health care provider assured your privacy during the examination	364 (86.7)	56 (13.3)
Was waiting time fair for examination.	281 (66.9)	139 (33.1)
Did the health care provider treated you compassionately and respectfully during ANC follow up	350 (83.3)	70 (16.7)
Were you involved in decision making as much as you want	334 (79.5)	86 (20.5)
Did you have well informed and good communication with the staffs	393 (93.6)	27 (6.4)
Did you received individualized care during the ANC visit	407 (96.9)	13 (3.1)
Did health care provider promotes partner/accompany during ANC	379 (90.2)	41 (9.8)
Are you happy with all the services you have got today	351 (83.6)	69 (16.4)

## Factors associated with respectful focused antenatal care

The result of multivariate analysis showed that respondent’s level of education, the status of pregnancy, gravidity, and average family monthly income were some of the factors associated with respectful focused antenatal care at *p* value < 0.05.

Respondents who had no formal education were 8.3 [AOR = 8.3 (95%CI 9.15–17.47)] times more likely to experience abuse during FANC than those respondents who have an educational level of collage and above. Similarly, respondents who had educational levels of primary and secondary were 2.45[AOR = 2.45 (95%CI 1.07–5.62)] times more likely to experience abuse during FANC than those respondents who had an educational level of collage and above.

Respondents who had an average family monthly income of less than or equal to 3000 Ethiopia birr were 3.16 [AOR = 3.16 (95%CI 1.52–6.57)] times more likely to experienced abuse during FANC as compared to those respondents who had an average family monthly income of greater than 3000 Ethiopian birrs. Respondents who had unplanned pregnancies were 9.9 [AOR = 9.90 (95%CI 3.48–8.16)] times more likely to experience violence during FANC as compared to those respondents who had planned pregnancy. Respondents who had multigravida were 8 [AOR = 8.82 (95%CI 2.90–6.80)] times more likely to have reported abuse during FANC than those respondents who had primigravida (Table 4).

**Table 4:** Factors associated with respectful focused antenatal care at Shashemene town hospitals, 2019

	Abuse during FANC		COR	AOR
	Yes	No		
Age				
20–25	152	29	1	1
26–30	155	69	2.33(1.43–3.80)*	3.97(1.73–9.12)
≥ 31	9	6	–	–
Mothers level of education				
No formal education	137	57	3.62(1.88–6.94)*	8.3(9.15–17.47)*
Primary and secondary	72	28	3.38(1.64–6.95)*	2.45(1.07–5.62)*
College and above	113	13	1	1
Average family monthly income				
≤ 3000	195	42	1	1
>3000	127	56	2.04(1.29–3.24)*	3.16(1.52–6.57)*
Family size				
1–3	129	28	1	1
>3	193	70	1.67(1.02–2.73)*	0.16(0.63 − 0.43)
Residency				
Urban	171	56	1	1
Rural	151	42	1.17(0.75–1.85)	0.12(0.04–0.31)
Status of pregnancy				
Planned	308	80	1	1
Unplanned	14	18	4.95(2.36–10.38)*	9.90(3.48–8.16)*
History of abortion				
Yes	28	5	1	1
No	293	93	1.71(0.66–4.72)	0.98(0.14–6.65)
History of stillbirth				
Yes	29	5	1	1
No	293	93	1.84(0.69–4.89)	4.72(0.67–12.95)
Gravidity				
Primgravida	100	14	1	1
Multigravida	222	14	2.70(1.46–4.99)*	8.82(2.90–6.80)*

*Significant with P < 0.05

## Discussion

This study was conducted to assess respectful focused antenatal care and associated factors among pregnant women who visit Shashemene town public hospitals, Oromia Ethiopia; we found that 37% of participants were committed abusive care during antenatal care. This finding suggests that health care providers should take into account the potential risk of abuse while assessing clinical health assessment during antenatal care visits, childbirth, and post-natal visits of a woman. Besides, for healthcare planners this is vital. This knowledge can be used to build relevant programs, channeling scarce resources to teaching what is needed as opposed to imparting messages that are already known. This level of abusive care during antenatal care is higher than what is reported from a study done elsewhere in Ethiopia [14, 15]. The discrepancy of these findings might be attributed to the difference in method used and study settings, socio-demographic characteristics of the study participants, and the availability and accessibility of the health services infrastructures. On the other hand; the report in this study implies that there is a lack of balance that the zone health office and regional health bureau could work in collaboration with the local health caregiver to lessen the abusive care of women during antenatal care.

This finding was lower than the study conducted in Enugu Nigeria, Pakistan, and other parts of Ethiopia [11–13]. This difference can be explained by the difference in the background of the study participants and the time gap. In addition to this, the health system-related factors might contribute to this difference due to the extensive work of health extension workers and various health care institutions in awareness creation about the drawback of abusive care in the study area. The other possibilities might be due to the difference in socio-economic and study participants since the previous study was conducted among laboring mothers where labor pain by itself may make the mother not perceive whether they were respected or not at the time of pain. The finding implies that there is crated platform for maternity care providers that could help them to be aware of local values, beliefs, and traditions to anticipate and meet the needs of women, gain their trust and work with them.

In this study, we have found some factors associated with abusive care during the antenatal period.

These included having no formal education, low average monthly family income, having an unplanned pregnancy, and being multigravida. Respondents who had no formal education were almost 8 times more likely to experience abuse during FANC than those respondents who had an educational level of college and above. Similar studies conducted in Ethiopia have found that education level is an important factor of abusive care during antenatal care [14]. This might be explained as women who did not attend formal education could not easily understand the drawback of abusive care on the health of the women themselves. Moreover, women who had no formal education will not have better awareness, understanding of birth preparedness, and complication readiness plan which was informed by health care providers.

Respondents who had an average family monthly income of less than or equal to 3000 Ethiopian birrs were almost 3 times more likely to experienced abuse care during FANC as compared to those respondents who have a family monthly income of greater than 3000 Ethiopian birrs. This finding is consistent with the study conducted in other parts of Ethiopia [12]. This might be because those women who had low average monthly income will not have better access/contact with the healthcare provider in the health facility within the recommended ANC period. Which in turn the women will not have information about the effect of abusive care on the health of the women themselves and their infants in the mother to child health (MCH) clinic; these delaines may make themselves not to be respected secondary to dissatisfaction. Similarly, those respondents who had unplanned pregnancies were almost 10 times more likely to experience violence during focused antenatal care than those respondents who had planned pregnancies. This might be due to unplanned pregnancy might not be usually supported; as a result of this, pregnant women might not be respected by their partners and they will not receive early and adequate prenatal care than those with planned pregnancy.

### Limitation

This study was a cross-sectional study and may not show the cause and effect relationship. The other limitation could be a small sample that might lead to statistical imprecision. We also didn’t find sufficient data to compare and contrast respectful focused antenatal care with others, so it makes our discussion shallow.

## Conclusions

This study depicted that the prevalence of abusive focused antenatal care was 63%. Having no formal education, low family monthly income, having an unplanned pregnancy, and being multigravida were some of the factors associated with respectful focused antenatal care, so health care providers should treat all maternity care users regardless of maternal educational status and economic background. Besides providing respectful, individualized, and client-centered care for all women at every contact is very important to attract more mothers to the health facility.

## Supplementary information

**Additional file 1**. Qustionery.

## Data Availability

The full data set and other materials about this study can be obtained from the corresponding author on reasonable request.

## Abbreviations

ANC: Antenatal care
AOR: Adjusted odds ratio
CI: confidence interval
FANC: Focused antenatal care
SPSS: Statistical package for social sciences

## References

[1] Secretariat C. Ensure healthy lives and promote well-being for all, at all ages (SDG 3). 2017.

[2] Organization WH (2015). Levels and Trends in Child Mortality: Report 2015: Estimates Developed by the UN Inter-Agency Group for Child Mortality Estimation (IGME).

[3] WHO, U, UNFPA. World Bank Group, and the United Nations Population Division. Trends in maternal mortality: 1990 to 2015. Estimates by WHO, UNICEF. 2015.

[4] WH Organization, Unicef, UNFPA/World Bank (2010). Trends in maternal mortality: 1990 to 2008.

[5] Ethiopia E, Demographic, Health Survey. 2016. 2012: ICF International, central Statistical Agency, July 2017.

[6] Organization WH. WHO recommendations on antenatal care for a positive pregnancy experience: World Health Organization; 2016. 28079998

[7] Freedman LP, Kruk ME (2014). Disrespect and abuse of women in childbirth: challenging the global quality and accountability agendas. Lancet.

[8] Lincetto O, Mothebesoane-Anoh S, Gomez P, Munjanja S. Antenatal care. In: Opportunities for Africa’s newborns: practical data, policy and programmatic support for newborn care in Africa. 2006, p. 55–62.

[9] Council RMCA. White ribbon alliance for safe motherhood (2011). Respectful maternity care: the universal rights of childbearing women Washington, DC. WRA Retrieved (August 8th, 2013), p. 1–6.

[10] Reed R, Sharman R, Inglis C (2017). Women’s descriptions of childbirth trauma relating to care provider actions and interactions. BMC Pregnancy Childbirth.

[11] Okafor II, Ugwu EO, Obi SN (2015). Disrespect and abuse during facility-based childbirth in a low‐income country. Int J Gynecol Obstet.

[12] Gebremichael MW, Worku A, Medhanyie AA, Berhane Y (2018). Mothers’ experience of disrespect and abuse during maternity care in northern Ethiopia. Global Health Action.

[13] Azhar Z, Oyebode O, Masud H (2018). Disrespect and abuse during childbirth in district Gujrat, Pakistan: a quest for respectful maternity care. PloS ONE.

[14] Asefa A, Bekele D (2015). Status of respectful and non-abusive care during facility-based childbirth in a hospital and health centers in Addis Ababa,&nbsp;Ethiopia. Reprod Health.

[15] Nawab T, Erum U, Amir A, Khalique N, Ansari MA, Chauhan A (2019). Disrespect and abuse during facility-based childbirth and its sociodemographic determinants–A barrier to healthcare utilization in rural population. J Family Med Primary care.

