# Supplementary material for: Respectful focused antenatal care and associated factors among pregnant women who visit Shashemene town public hospitals, Oromia region, Ethiopia: a cross‐sectional study
Source: BMC Womens Health. 2021 Mar 4;21:92. doi: 10.1186/s12905-021-01237-0 (PMC7934531; doi:10.1186/s12905-021-01237-0)
Supplement: Supplementary file 1 — Additional file 1. Qustionery. [file 12905_2021_1237_MOESM1_ESM.docx]

Table1: An English version Questionnaire to assess Respectful focused antenatal care and associated factors among pregnant women who visit Shashemene town public hospitals, Oromia region, Ethiopia 2019.

| **S.no** | **Question** | | | **Response** | | **Skip** |
| --- | --- | --- | --- | --- | --- | --- |
| **Part I: Socio-demographic characters of mother** | | | | | | |
|  | Age of mother in completed years | ------------- | | | |  |
|  | Marital status | 1. Single 2. Married 3. Divorced 4. Widowed | | | |  |
|  | Mother‘s religion | 1. Orthodox 2. Protestant 3. Muslim 4. Other | | | |  |
|  | Ethnicity | 1. Oromo 2. Amhara 3. Tigre 4. Others (specify)… | | | |  |
|  | Residency | 1. Urban 2. Rural | | | |  |
|  | Mother‘s level of education | 1. No formal education 2. Primary (1-8) 3. Secondary (9-12) 4. Collage and above | | | |  |
|  | Mother’s occupation | 1. House wife 2. Private employee 3. Government employee 4. Merchant 5. Other | | | |  |
|  | Husband educational status | 1. No formal education 2. Primary (1-8) 3. Secondary (9-12) 4. Collage and above | | | |  |
|  | Average family’s monthly income. In ETB birr? | ____________________ | | | |  |
|  | Family size | __________________ | | | |  |
| **Part II: Obstetrics Characteristics** | | | | | | |
|  | Gravidity | | _________________ | | |  |
|  | Parity | | ___________________ | | |  |
|  | History of abortion | | 1. Yes 2. No | | |  |
|  | History of still birth | | 1. Yes 2. No | | |  |
|  | History of institutional delivery | | 1. Yes 2. No | | |  |
|  | Do you have history of ANC visit for the previous pregnancy? | | 1. Yes 2. No | | |  |
|  | Status of pregnancy | | 1. Planned 2. Unplanned | | |  |
|  | Frequency of Current FANC visit | | -------------------_____________ | | |  |
|  | Do you have a plan to give birth in a health facility's | | 1. Yes 2. No | | |  |
|  | If no why (n=40) | | Home is better | | |  |
|  |  |  | Fear of health care providers | | |  |
|  |  |  | Not satisfied with antenatal care | | |  |
|  |  |  | No reason | | |  |
| **Part III:** Respectful/client-centered items during the focused antenatal care visit | | | | | **Response** | |
|  | Did the health care provider respect your culture and religion during the general examination | | | | 1. Yes 2. No | |
|  | Did the health care provider explain procedures by giving a greeting before the examination | | | | 1. Yes 2. No | |
|  | Did the health care provider treated you in a friendly manner | | | | 1. Yes 2. No | |
|  | Did the Health care provider showed his/her concern and empathy | | | | 1. Yes 2. No | |
|  | Did the Health care provider explained types of laboratory investigation in a satisfactory way | | | | 1. Yes 2. No | |
|  | Did health care provider caring for you with a kind approach by calling your name | | | | 1. Yes 2. No | |
|  | Did the health care provider responded to your needs whether or not you asked during counseling on birth preparedness and complication readiness | | | | 1. Yes 2. No | |
|  | Did the health care provider assured your privacy during the examination | | | | 1. Yes 2. No | |
|  | Was waiting time fair for examination. | | | | 1. Yes 2. No | |
|  | Did the health care provider treated you compassionately and respectfully during ANC follow up | | | | 1. Yes 2. No | |
|  | Were you involved in decision making as much as you want | | | | 1. Yes 2. No | |
|  | Did you have well informed and good communication with the staffs | | | | 1. Yes 2. No | |
|  | Did you received individualized care during the ANC visit | | | | 1. Yes 2. No | |
|  | Did health care provider promotes partner/accompany during ANC | | | | 1. Yes 2. No | |
|  | Are you happy with all the services you have got today | | | | 1. Yes 2. No | |

**Thank you in advance!!!**
